# The Health Cost of Organizational Citizenship Behavior: Does Health-Promoting Leadership Matter?

**DOI:** 10.3390/ijerph19106343

**Published:** 2022-05-23

**Authors:** Bo Fu, Jian Peng, Tao Wang

**Affiliations:** 1 School of Management, Guangzhou University, Guangzhou 510006, China; dr.fubo613@gmail.com or; 2 Office of Finance, Sun Yat-sen University, Guangzhou 510275, China; wangtao39@mail.sysu.edu.cn

**Keywords:** organizational citizenship behavior, citizenship fatigue, health complaints, health-promoting leadership

## Abstract

Previous research has mainly focused on the positive effects of organizational citizenship behavior (OCB). This study questions the positive impact of OCB, arguing that there is a health cost of OCB. Based on the conservation of resource theory, this study expects that OCB triggers citizenship fatigue, which, in turn, negatively affects employees’ health and results in health complaints. This study also seeks to find a moderator (health-promoting leadership) that could mitigate the negative effects of citizenship fatigue (caused by engaging in OCB) on health complaints. To test our predictions, we collected three-wave data from 207 leader–subordinate dyads. The results of regression analyses show that OCB is positively related to employees’ health complaints, which is mediated by citizenship fatigue. Health-promoting leadership weakens the positive relationship between citizenship fatigue and health complaints, thus negatively moderating the indirect relationship between OCB and health complaints via citizenship fatigue. This study provides theoretical and practical implications for future research directions.

## 1. Introduction

Organizational citizenship behavior (OCB) is defined as an individual act which is not directly or definitely recognized by the formal reward system but that has been proven conducive to the efficient and effective operation within organizations [1]. It was initially conceptualized purely as a discretionary act [2,3] and has been found to exert various beneficial outcomes (e.g., performance and wellbeing) for organizations and employees themselves [3,4,5,6]. However, some studies point out that employees engage in OCB because this behavior is often required as part of their job [7,8]. In order to reconcile the different understandings of OCB, Organ (1997) [9] considered that individual behavior need not be discretionary to be considered an act of citizenship. He modified the definition of OCB as a broad act that contributes to the “organizational context that supports task performance” (1997, p. 91).

Consistent with Organ (1997) [9], existing research found that employees often engage in OCB not out of their discretion but because they feel compelled to do [10,11]. Research has shown that employees often engage in OCB because they feel pressure from their organization [12] or to avoid punishments [13]. Many employees view OCB as part of their job responsibilities [14,15]. In some cases, employees engage in OCB not because they want to but because they have to [10].

Although a growing body of research recognizes that employees are often externally compelled to engage in OCB, the negative consequences of excessive engagement in OCB have not been well explored. Indeed, OCB requires individuals to invest cognitive, emotional, and physical resources in activities that go beyond the demands of their job duties [16], and when these resources are scarce, the burden of participating in OCB can create an internal tension that depletes an individual’s internal resources and leads to fatigue [10,15,17,18,19,20,21]. A few scholars have theorized that citizenship fatigue occurs when employees must engage in OCB [16,22,23,24]—suggesting the potential drawbacks of OCB for health. However, to our knowledge, little empirical research has examined a negative causal relationship between OCB and employee health and how to mitigate the negative consequences of OCB on employee health. Accordingly, we draw on the conservation of resource theory (COR) [25,26] to consider how OCB may lead to subsequent employee health complaints via citizenship fatigue and how to make up for the personal health cost that OCB incurs.

The present study explores the relationship between OCB and health complaints. As mentioned above, OCB has a cost. It consumes cognitive and emotional resources and physical strength [10,21]. which can lead to tiredness, exhaustion, and boredom if one engages in it for long, i.e., citizenship fatigue. Bolino et al. (2015) [16]. defined the citizenship fatigue as a psychological experience of boredom, fatigue, or exhaustion caused by one’s frequent engagement in OCB. The COR theory holds the view that individuals are naturally inclined to acquire or conserve valuable resources. In this respect, people will experience both psychological and physiological discomfort when they perceive resource losses in the work environment [25]. Therefore, employees who frequently engage in OCB tend to perceive that their resources are threatened, for which they may suffer from psychological and physical fatigue that can lead to physical and psychological health problems and make health complaints.

Another research question of this study is how to make up for the personal health cost that OCB incurs. To reduce the health complaints caused by citizenship fatigue, this study approaches the way of reducing the negative influence of OCB from the perspective of leadership behavior. The COR theory believes that individuals can replenish depleted resources by acquiring internal or external ones to alleviate the negative impact so caused [25]. When employees suffer from fatigue caused by OCB, i.e., citizenship fatigue, the resulting health complaints can be reduced if their leaders can timely notice them and take corresponding action to maintain and improve their health, i.e., health-promoting leadership (HPL) [27]. In this respect, the present study deduces that even if individuals frequently engage in OCB, their health complaints caused by citizenship fatigue can be alleviated by HPL, for HPL can replenish their personal resources.

Based on the above discussion, the present study aims to deepen the current research on the negative impacts of OCB by exploring its relationship with health. To be more specific, this research examines the health cost of individuals who engage in OCB and reveals the important role that HPL plays in mitigating the cost. To achieve the goal, it needs to complete the following two steps. Firstly, it examines how OCB can positively affect employee citizenship fatigue, which leads to employee health complaints as a consequence. Employees’ engagement in OCB can propel them to consume a great deal of individual resources [16,19,21,28]. Therefore, this study researches the possible correlation between OCB and citizenship fatigue from the perspective of the COR theory [25,26], for citizenship fatigue may result in a substantial amount of resource consumption, thus negatively affecting employees’ health and causing health complaints.

Secondly, the current study introduces HPL as a moderator among these relationships given that the existing research has confirmed leadership qualities as an important explanatory psychosocial factor with regard to employee health [29]. For instance, Gurt et al. (2011) [30] argue that leaders’ clear consideration and active involvement in terms of subordinates’ health can bring benefits to employees. As discussed above, it is likely that HPL alleviates the effect of employee citizenship fatigue on health complaints. Thus, this study holds the view that OCB may lead to growing citizenship fatigue and health complaints, but such a relationship may be weakened by the leadership that shows care about employee health.

**Figure 1:**
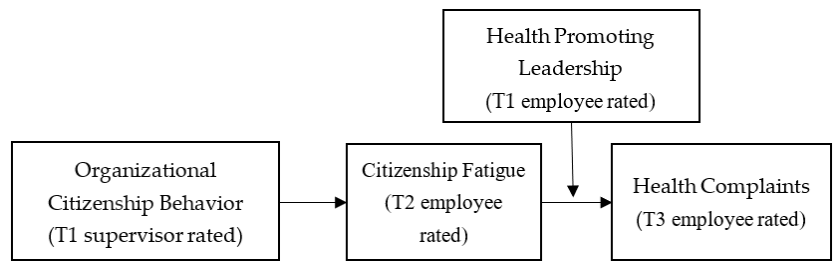
Hypothesized theoretical model.

Consequently, this study contributes to the research in this field in three ways. First of all, it enriches the study on the negative impact of OCB through determining how it has an adverse effect on employee health complaints and fatigue. Moreover, it draws on the COR theory to illustrate that OCB consumes resources, thus affecting employees’ health. Secondly, this study proposes that citizenship fatigue serves as a bridge that connects OCB with health complaints—a theoretically reasonable research gap that has gained insufficient attention up to now. Thirdly, this research investigates the way to lower employees’ health costs incurred by their engagement in OCB. Therefore, the present research focuses on how HPL moderately affects the relationship between OCB and health complaints. To achieve the goal, it constructed a moderated intermediary model, as shown in Figure 1, in which HPL conditionally affects the indirect effect of OCB on health complaints, which is mediated by citizenship fatigue. In addition, this study tested the theoretical model by using the field data from multiple sources over three time slots.

## 2. Theory and Hypotheses

### 2.1. The Health Cost of OCB

#### 2.1.1. OCB and Citizenship Fatigue

The COR theory [25,26] presumes that people have certain motivations to gain and protect things they treasure, such as objects, situations, personal traits, time, and energy, i.e., resources. In the case that their resources are lost or at risk of being lost or depleted, they are most likely to suffer from stress or burnout. Considering that employees who engage in OCB are usually restrained by resources [19], this study explains the negative impact of one’s engagement in OCB from a resource depletion perspective with the COR theory as its theoretical underpinning; it argues that a high level of OCB is correlated with citizenship fatigue because OCB results in resource consumption.

Citizenship fatigue refers to “the state of exhaustion, weariness, or tension that employees feel as a result of getting involved in OCB [16]. Current research has suggested that subordinates who suffer from citizenship fatigue will feel depressed or undervalued. Citizenship fatigue arises from employees’ engagement in OCB, for it consumes their energy or personal resources [16]. Henceforth, when employees who obtain negative feedback about OCB, such as their being pushed to engage in excessive OCB, employees are susceptible to high levels of citizenship fatigue.

In light of the COR theory, employees should invest resources in the case that they perform OCB; in other words, their personal resources are reduced and even depleted in this process [21]. OCB involves the activities that go above and beyond employees’ job duties (e.g., assisting colleagues to lighten their workload and listening to coworkers’ problems). As a consequence, when employees engage in OCB, they consume their personal resources, especially their time and energy. In this aspect, current research has confirmed that engaging in OCB causes resource consumption, which leads to working pressure [18] and tiredness [16]. Therefore, this study holds the view that employees’ engagement in OCB consumes resources, thus resulting in citizenship fatigue.

Recent research has investigated the negative consequences of OCB [31,32]. OCB can deplete excessive resources given that it diverts employees from in-role responsibilities and places great pressure on employees by requiring them to take multiple role responsibilities simultaneously [31]. Likewise, Banwo and Du (2020) [32] found that OCB incurs personal costs for the reason that it demands employee’s working resources. Therefore, citizenship fatigue is most likely to occur if there is no sufficient replenishment of resources after one performs OCB, according to the COR theory [25,26].

In general, employees performing OCB are likely to experience citizenship fatigue, as the heavy workload brought by many job roles can scramble for the same units of time and energy within an existing resource pool [16,21]. Consequently, citizenship fatigue may increase in the employees who allocate resources regularly for ongoing participation in OCB activities, which has been supported by previous research. For example, it has been found that a motivation to be a good citizen and perform OCB will lead to an additional investment into working effort and energy, which can lead to negative results, such as role overload, job stress [18], and emotional exhaustion [21]. Therefore, subordinates who frequently spend much time performing OCB tend to suffer from higher levels of citizenship fatigue. Based on the above practical and theoretical bases, the following hypothesis is proposed:

**Hypothesis** **1.** *OCB has a positive correlation with citizenship fatigue.*

#### 2.1.2. Citizenship Fatigue and Health Complaints

The specific situation in which employees are confronted with citizenship fatigue still remains to be explored. Citizenship fatigue means a state featured by affect and cognition alike. In this respect, it is one’s affective and cognitive feeling about OCB. Since OCB leads employees to perform the tasks that are beyond their work requirements, it consumes their internal resources [18]. In other words, excessive OCB brings about tiredness and exhaustion, i.e., citizenship fatigue. Once employees experience citizenship fatigue, they often tell themselves they have had “enough” or “have got tired of this” [33]. Previous studies have proven that negative emotions such as perceived role strain and daily stressors (hassles) can lead to subjective health [34]. Subjective health complaints (HC) refer to a term which describes “unexplained symptoms” [35], i.e., relatively vague complaints on personal health, with or without a defined diagnosis, such as headache, backache, nervousness, and insomnia [36]. Technically speaking, although these health complaints are not exactly diseases, they could still have a negative effect on employees’ performance and engagement in both working life and social activities [37].

In the workplace, employees usually perform their job tasks. In this case, their engagement in OCB requires them to make extra efforts that are beyond expectations in a limited time. OCB consumes their energy and time, thus resulting in fatigue. According to the COR theory, individuals experience both physical and psychological discomfort when they perceive resource losses in the work environment, both anticipated and actual ones, or when they cannot replenish their resources after consuming them [25]. For employees, excessive involvement in OCB incurs time costs and consumes physical and psychological resources, which leads to citizenship fatigue, thus causing psychological and physical discomfort [38] and even health complaints. For instance, when employees work overtime excessively or often share their colleagues’ work, they will experience negative feelings such as weariness and exhaustion. The negative emotions are not helpful to their physical and psychological health and can result in health complaints, both physiological (headache, muscle pain, gastrointestinal problems, etc.) and psychological ones (anxiety, depression, irritability, etc.) [39]. Based on the above discussions, this study puts forward the following hypothesis:

**Hypothesis** **2.** *Citizenship fatigue exerts a positive impact on employee health complaints.*

#### 2.1.3. The Mediating Role of Citizenship Fatigue

Hypothesis 1 predicts a positive correlation between OCB and citizenship fatigue, whereas Hypothesis 2 forecasts a negative correlation between citizenship fatigue and subjective health complaints. These two hypotheses point out a theoretical model in which OCB indirectly reduces subjective health complaints by giving rise to citizenship fatigue, which is consistent with the COR theory [25,26] and the previous study on citizenship fatigue [16], which suggests that a high level of OCB dramatically consumes personal resources, thus causing pressure or negative reactions to pressure that will further result in mental and physical health problems. OCB may directly induce employees’ health costs, so this study argues that it is the experience of citizenship fatigue that will incite mental and physical health problems in employees. On the other hand, employees may remain energized after performing OCB, which suggests that their resource consumption does not make them feel exhausted [19] unless their fatigue reaches the critical point. Therefore, this research identifies citizenship fatigue as the tipping point that triggers the decline of employees’ health. In other words, employees show psychological and physical symptoms only when they are exhausted due to their performing OCB.

As mentioned above, OCB is a behavior beyond employees’ job responsibilities [16]. It has been proven that OCB can be beneficial to individual and organizational performance in a number of studies, but its positive impact comes at the expense of employees’ health. If they frequently engage in OCB, and even regard it as part of their daily work, they have to consume their internal resources such as time, energy, and physical strength. At this point, employees will realize that they are facing the risk of resource losses or actually suffer them, such as prolonged working hours or additional activities, which sustains further losses [40]. Faced with the resource losses caused by extra work or increasing work load, individuals will have a negative experience of physical and mental fatigue, thus causing health complaints [37,41]. In other words, when OCB causes citizenship fatigue, employees will have concerns about their physical and mental conditions, thus making health complaints. Based on the above discussion, this study deduces that citizenship fatigue plays a mediating role in the relationship between OCB and health complaints. OCB leads employees to experience resource losses at work, including the threat of resource losses that they perceive and the actual losses that they suffer, or it makes employees unable to replenish the resources that they have invested. Employees will feel tired due to resource depletion, and eventually experience citizenship fatigue, a negative feeling that triggers physical and mental health problems and then health complaints. Therefore, this research puts forward another hypothesis, as shown below:

**Hypothesis** **3.** *Citizenship fatigue mediates the relationship between OCB and health complaints.*

### 2.2. The Moderating Role of Health-Promoting Leadership

Leadership is the most important source of information for employees and thus influences the way employees perceive and perform their work [42,43,44]. To facilitate employees’ positive job attitude and personal growth, leaders are encouraged to provide support for employees, such as expressing concern, providing personalized mentoring, and taking into account the needs of employees [44,45]. When they perceive a high level of leader support, the physical and cognitive resources employees devote to achieve the leader’s vision can be restored. Hence, perceived leader support is related to increased work effort and greater job involvement of employees, which facilitate employees’ satisfaction, positive function, performance, and personal [42,46,47,48]. Given the importance of leader support, we consider a healthy specific leadership support, HPL, that alleviates employees’ health complaints after experiencing citizenship fatigue.

The COR theory also recognizes the role of leadership in shaping employees’ resources. The COR theory assumes that pressure is less likely to arise if people possess resources which can help them deal with stressors and challenges in their lives. Its principle of resource acquisition holds that employees can alleviate internal resource losses by obtaining external resources to replenish the consumed ones [25]. Herein, in addition to something inherent in a person’s work, resources can also be granted by a supervisor [49]. This serves as a theoretical basis for how to alleviate the health cost incurred by OCB. When employees pay health costs incurred by OCB, it is of great importance to acquire external resources to replenish the consumed ones. Consequently, this study introduces a leadership strategy that focuses on employee health, i.e., HPL, an external resource that can help alleviate the negative impact of OCB on health complaints caused by citizenship fatigue. This study adopts HPL because it involves a behavioral process in which leaders take actions to maintain and improve employees’ health [30], which can alleviate health complaints that result from citizenship fatigue.

How can people alleviate or prevent employee health complaints when they are exhausted because of OCB? Citizenship fatigue causes health complaints based on the premise that employees cannot replenish their resources after using part of them, and accumulated fatigue causes complaints about both physical and mental health. In this respect, employees need to acquire resources so as to reduce their citizenship fatigue and subsequent impacts. The COR theory holds the view that individuals can replenish their physical and mental resources and alleviate the negative impact of resource losses by acquiring external resources. Among the external resources, the most important one is health support from the leadership [50], i.e., HPL. It is a leadership strategy focusing on employee health and also a unique set of leadership behaviors which can impact employees’ health [30]. It is also defined as a behavioral process in which leaders take action to maintain and improve employees’ health [27]. HPL enables employees to know how to stay fit and lead a healthy life, so as to help them achieve and maintain their mental and physical health [51]. That means leaders can affect the health of their employees by emphasizing the importance of fitness in the workplace [52].

At present, HPL has been proven to have positive effects. HPL can reduce employees’ stress and job burnout, which helps them improve their health and wellbeing [53]. Moreover, HPL facilitates reducing their absenteeism and medical expenses, thus increasing their productivity [54]. HPL describes the extent to which employees feel their health is cared about by their leaders [55]. Healthy working methods and health-related external resources (i.e., relaxation and stress relief) that leaders provide to employees will reduce their health complaints, which result from citizenship fatigue. In this respect, even if they frequently engage in OCB, leaders can adopt HPL to help replenish their internal resources, thus reducing their health complaints caused by citizenship fatigue. On the contrary, if leaders ignore or show indifference to their health problems, they will acquire no external resources to make up for the lost ones. This way, their health problems cannot be alleviated, thus causing frequent health complaints. That is why the following hypothesis has been proposed:

**Hypothesis** **4.** *HPL plays a negative moderating role in the relationship between citizenship fatigue and health complaints, which means the higher the level of HPL is, the weaker the positive correlation between citizenship fatigue and health complaints, and the lower the HPL, the stronger the correlation between them.*

Based on Hypotheses 3 and 4, the present study constructs a moderated mediation model. In this model, HPL negatively moderates the mediating role that citizenship fatigue plays in the relationship between OCB and health complaints, which means that the mediating effect of citizenship fatigue depends on HPL. The COR theory believes that external resources can alleviate the negative results caused by resource losses, such as the additional workload resulting from the engagement in OCB [25]. As a way of providing employees with external resources, HPL is effective in alleviating health complaints that are caused by OCB via citizenship fatigue. Specifically, HPL helps employees improve their health in multiple ways: (1) creating healthy working conditions, including avoiding unilateral posture and providing sufficient space and bright working environment [56,57], (2) supporting and encouraging employees to participate in the activities that improve their occupational health, such as shoulder and neck training, relaxation, and stress management [58,59], and (3) leading them to have a healthier lifestyle, such as having a healthy diet, quitting smoking, and taking physical exercise [30,52]. These methods can help employees replenish their physical and psychological resources in time after performing OCB and experiencing exhaustion [54], so as to reduce the risk of employees’ making health complaints [55]. Therefore, this research puts forward another hypothesis, as follows:

**Hypothesis** **5.** *HPL negatively moderates the mediating role that citizenship fatigue plays in the relationship between OCB and health complaints. The more HPL, the weaker the mediating effect that citizenship fatigue exerts on the relationship between OCB and health complaints, and the less HPL, the stronger the mediating effect of the citizenship fatigue on the correlation.*

## 3. Method

### 3.1. Participants and Procedure

This research collected data using the questionnaire method. Its respondents were the employees of eight enterprises based in the Pearl River Delta of South China, which involve different industries such as electronic manufacturing, real estate, and media. Each enterprise recruited volunteers from their employees to fill in the questionnaire. Before the questionnaire had been issued to the respondents, they were informed that the data collected from the questionnaire they were to fill out would only be used for academic research, that these questions had no right or wrong answers, and that their personal information would not be disclosed to ensure the anonymity and confidentiality of the questionnaire. To avoid the serious impact of common method biases on the results, this study collected the questionnaire data in three time periods, with an interval of two months. The first stage collected the data about evaluations from oneself and others. The self-evaluation part required the respondents to fill in demographic information and HPL data, while the other part was about the comment on the OCB that respondents engaged in, which was evaluated by their immediate superiors. In the second phase, the respondents were asked to fill in the scales that measured their citizenship fatigue, emotional commitment, and negative emotions. In the third stage, they were asked to evaluate their own health complaints.

In this study, a total of 252 questionnaires were distributed, of which 207 ones were gathered. In the first stage, 252 questionnaires were distributed while 230 valid ones were obtained. In the second stage, 230 questionnaires were distributed, of which 217 valid ones were collected. In the last phase, 217 questionnaires were distributed, and 207 valid ones were recovered. In terms of gender, male respondents (52.7%) were slightly more than female ones. Regarding age, the participants were mainly aged between 21 and 30 years old (45.4%), which was followed by those aged between 31 and 40 years old (31.4%), and those aged 51 years old and above accounted for a small proportion (6.8%). With regard to education, the respondents with bachelor’s degrees or above accounted for 56%. As for working hours per week, more than half of them (i.e., 58.5%) worked 35 to 45 h a week. In terms of seniority, the majority of them had worked for no more than 5 years or worked under the current immediate superior for no more than 5 years, accounting for 57.4% and 74.2%, respectively.

### 3.2. Measures

(1) Organizational citizenship behavior (OCB): The scale that Bolino et al. (2015) [16] used in research was adopted. It consists of 28 questions, of which seven items are about helping behavior, such as “The employee volunteers to work for the Department”, six items are about voice behavior, such as “The employee proposes suggestions on the problems that lower the work efficiency of the whole Department”, and the remaining 15 ones are about personal initiative, such as “The employee works overtime on a voluntary basis”.

(2) Citizenship fatigue: The seven-question scale developed by Bolino et al. (2015) [16] was adopted to assess this construct, which includes items such as “The work task goes beyond the job responsibility, which often makes me exhausted”.

(3) Health complaints: The 12-question scale compiled by Moksnes et al. (2011) [60] was used to measure health complaints in this research. It consists of two aspects, i.e., physical and psychological. The items that measure psychological health complaints include “nervousness” and “irritability”, while those that measure physical ones involve “a cold” and “stomachache”.

(4) Health-promoting leadership (HPL): This study adopted a seven-question scale to measure HPL. This scale includes items such as “My immediate superior urges me to pay attention to my physical health and often discuss with me about how to stay healthy both physically and mentally”.

(5) Control variables: To avoid the impact of demographic variables on the research results, this study set the following variables as controls—age, gender, education level, years of service, and years of working with immediate superiors. In order to distinguish citizenship fatigue from other emotional variables, this study also set the negative emotions of employees as a control variable using the scale developed by MacKinnon (1999) [61], such as “trembling with fear”.

### 3.3. Analytic Approach

In this study, descriptive statistics and correlation analysis of the main variables were generated using SPSS 22.0 (IBM, New York, NY, USA), and a confirmatory factor analysis using Mplus 8.0 [62] was performed to test the discriminant validity of the variables. For hypothesis testing, path analyses were conducted using the Process 3.3 plug-in for SPSS 22.0, and the bias-corrected 95% confidence interval (CI) for indirect effect was estimated using the bootstrapping method.

## 4. Results

### 4.1. Results of Confirmatory Factor Analyses

Confirmatory factor analysis was used to test the validity of the following four variables: OCB, citizenship fatigue, health complaints, and HPL. It can be seen from Table 1 that all the fitness indexes of the hypothetical four-factor model were good (χ^2^/df = 2.67, RMSEA = 0.08, SRMR = 0.05, TLI = 0.92, CFI = 0.94), which is markedly better than the combined three-, two- and single-factor models. Therefore, there is a good discriminant validity among the variables in this study.

### 4.2. Descriptive Statistics and Correlations

The means, standard deviations, and coefficients that this study involved are all listed in Table 2. The research has the following findings: (1) OCB is positively correlated with citizenship fatigue (r = 0.30, *p* < 0.01), (2) OCB (r = 0.39, *p* < 0.01) and citizenship fatigue (r = 0.32, *p* < 0.01) have a positive correlation with health complaints, and (3) HPL has a negative association with health complaints (r = −0.34, *p* < 0.01). 

#### 4.2.1. Testing Results of Main Effects

Model 2 examined the impact of OCB on health complaints. As shown in Table 3, OCB is positively correlated with health complaints (r = 0.43, *p* < 0.01), indicating that the more OCB employees engage in, the more likely they are to make health complaints. Therefore, Hypothesis 1 is supported. 

#### 4.2.2. Mediating Role of Citizenship Fatigue

As shown in Table 3, Model 1 reveals a remarkable positive correlation between OCB and citizenship fatigue (r = 0.29, *p* < 0.001), so H1 is validated. Model 2 shows a marked positive correlation between OCB and health complaints (r = 0.43, *p* < 0.001). Model 3 tested the positive effect of citizenship fatigue on health complaints (r = 0.44, *p* < 0.001), so Hypothesis 2 is validated. Model 4 shows that with OCB and citizenship fatigue included in the model that has control variables, OCB still exerts a positive impact on employee health complaints (r = 0.34, *p* < 0.001). That demonstrates the mediating role that citizenship fatigue plays in the relationship between OCB and health complaints. Therefore, Hypothesis 3 is validated.

To further verify the mediating role of citizenship fatigue, this study used the bootstrapping method to repeatedly sample 5000 times to test whether citizenship fatigue has a significant mediating effect. The results showed that the mediating role of citizenship fatigue in the relationship between OCB and health complaints reached 0.09, and the corresponding 95% confidence interval was [0.03, 0.16], which excluded 0. Therefore, the mediating role of citizenship fatigue is proven significant once more. In other words, Hypothesis 3 is further supported.

#### 4.2.3. Moderating Effect

**Figure 2:**
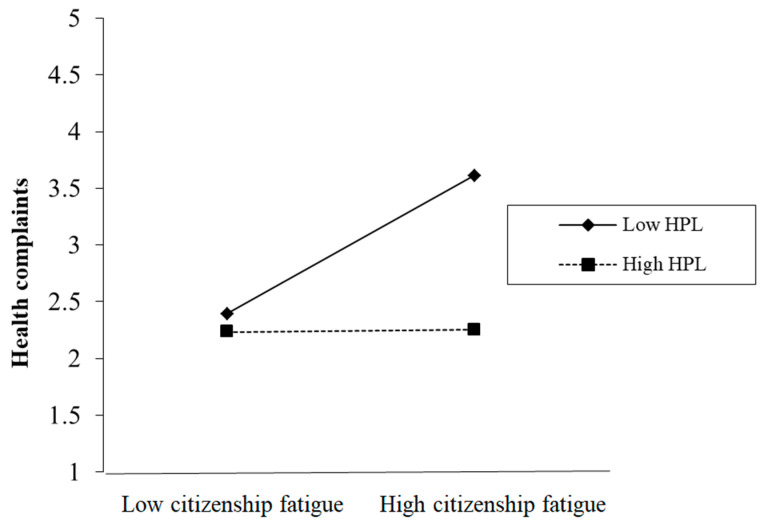
The moderating effect of HPL.

As listed in Table 3, Model 5 tested the moderating effect of HPL on the relationship between citizenship fatigue and health complaints. The corresponding outputs show that the interaction term of citizenship fatigue and HPL could negatively predict health complaints (*r* = −0.26, *p* < 0.001), which indicates that the health-promoting behavior of leaders weakens the positive impact of citizenship fatigue on health complaints. Based on the test results of Model 5 listed in Table 3, the moderating effect of HPL is shown in Figure 2. This way, Hypothesis 4 is validated. 

#### 4.2.4. The Moderated Mediating Effect

The path analysis was conducted to test the size and difference of the mediating effect that citizenship fatigue had when HPL was at different levels (i.e., high and low). Table 4 shows that when HPL is high, the indirect effect of OCB on employee health complaints via citizenship fatigue is −0.01, and the corresponding 95% confidence interval includes 0. On the other hand, when it is low, the indirect effect is 0.14, and the corresponding 95% confidence interval excludes 0. It can be seen that the differential values between different levels of HPL reach the level of significance, indicating that the higher the HPL, the weaker the mediating role that citizenship fatigue plays in the relationship between OCB and employee health complaints, and the lower the HPL, the stronger the mediating effect of citizenship fatigue. Therefore, Hypothesis 5 is verified.

## 5. Discussion

The present study re-examined the appropriate amount of OCB and the harm that excessive OCB may cause to employees. Based on the understanding of the negative effect of OCB [10], this study explored its negative impact from the perspective of the incurred cost that individuals may pay; this is also consistent with existing research findings that OCB triggers individual costs [21]. To achieve the purpose, this study draws on COR theory to explore how and when performing OCB at work can be costly. In line with the hypotheses that this study proposed earlier, it found that performing OCB is relevant to citizenship fatigue, which is related to health complaints. Furthermore, this study revealed the process in which the cost caused by OCB incurs and the corresponding compensation mechanism. Despite the fact that OCB can cause health problems, leaders’ health-promoting behavior helps make up for the health cost that OCB causes, which means that HPL has a moderating effect on the relationship between OCB and health complaints. Specifically, HPL negatively moderates the relationship between citizenship fatigue and health complaints and the indirect effect of citizenship fatigue on the relationship between OCB and health complaints. The higher the HPL is, the weaker the above relationships.

### 5.1. Theoretical Implication

Our research provides several theoretical implications for the field of OCB. First, considering that OCB is positive, existing research has generally focused on the positive outcomes OCB produces [33], however, recognizing OCB is likely to drain one’s resources, especially when employees realize that such behaviors are not discretionary but ought to be carried out. Therefore, researchers have called for studies that focus on the dark side of OCB [10,63]. In fact, it is a complicated phenomenon that individuals with limited time and energy engage in OCB and consume their resources. In this respect, Bergeron (2007) [19] referred to it as “the potential paradox of organizational citizenship behavior”. Therefore, our results extend prior work by revealing the health cost caused by OCB from the perspective of employees and how to make up for it.

Second, in revealing the dark side of OCB, the present study introduces the mediating role of citizenship fatigue. Bolino et al. (2015) [16] proposed the concept of “citizenship fatigue” by distinguishing it from burnout and stress in order to deeply explore the reaction that employees make if they turn OCB into their daily job. Moreover, they pointed out that employees with limited resources such as time and energy pay costs and experience fatigue if they engage in OCB. Based on their research, the present study managed to carry out in-depth research on the cost that OCB may cause to employees. It verifies that employees’ engagement in OCB leads to citizenship fatigue. Moreover, it further reveals the negative impact that OCB exerts on their physical and mental health, and in other words, that engaging in OCB can cause employee health complaints. Previous research focuses on the influence of OCB on organizational and job performance. However, this study shifted the focus to its impact on individuals, discussing the negative impact of OCB on individuals’ mental and physical health. To some extent, the present study approaches the appropriate amount of OCB; excessive OCB causes citizenship fatigue, and then leads to health problems.

Another theoretical contribution is how to mitigate the negative impact of OCB on employee health. Bergeron (2007) [19] and Bolino et al. (2015) [16] all have confirmed the negative impact of OCB, but they did not deeply reveal how to mitigate or make up for its adverse impact. Based on the COR theory, this study reveals the moderating role that HPL plays between citizenship fatigue and health complaints, and its moderating role in the mediation of citizenship fatigue between OCB and employee health complaints. Specifically, when leaders show less health-promoting behavior, employees’ perceived citizenship fatigue after engaging in OCB has a stronger positive impact on their health complaints. HPL, leaders’ attention to employees’ health, can function as the external resources that reduce the fatigue caused by OCB, which may lead to health problems later. Furthermore, it is also found that when leaders show a high level of HPL, the positive relationship between citizenship fatigue and health complaints will no longer be significant, which also fully proves the important role of HPL in making up for the cost OCB causes to employees.

### 5.2. Practical Implications

Enterprises attach great importance to the positive impact of OCB, which has been widely recognized by the academia. However, this study finds out that subordinates who perform OCB are likely to feel tired, which may threaten their physical and mental health. Consequently, organizations may face a dilemma: they hope that their employees actively engage in OCB to increase organizational benefits, but employees’ engagement in OCB may bring them negative effects, such as fatigue and threats to physical and mental health. The dilemma highlights the important role of organizational managers in maintaining their employees’ health.

Therefore, one primary problem that faces the organizations is how they can enable their organizational managers to give full play to HPL. Firstly, organizational managers should talk about health issues with employees and actively maintain and improve the working environment to make it beneficial to employee health. Secondly, organizational managers should correctly identify and balance their behaviors within and beyond their responsibilities; organizational managers are expected to establish a reasonable performance management system, salary system, and employee development system. In an era in which new technologies, industries, and models appear incessantly, the enterprises in the start-up and fast-growing stages need a large amount of OCB. Without fair, reasonable, and dynamically adjustable salary, performance management, and employee development systems, it will be difficult for their employees to continue performing OCB for a long time. To minimize employee citizenship fatigue, it is advised that the enterprises (1) restructure job duties and responsibilities, (2) timely adjust performance standards and salary structures and identify and compensate employees’ behaviors that are beyond their job tasks, and (3) incorporate OCB in their formal compensation system. This study helps the organizational management have a deeper understanding of the impact that OCB has on employees, thus guiding them on how to properly treat OCB. This way, employees can balance the relationship between their work tasks and OCB and then alleviate the cost incurred to individuals.

### 5.3. Limitations of the Current Study and Avenues for Future Research 

This research has some limitations. Firstly, the mediation of citizenship fatigue is one theoretical perspective that explains employee health problems caused by OCB, and there are other possible paths in which OCB influences employee health. In this respect, future research is advised to further discuss other potential influence paths. Secondly, this study focuses on the negative impact of OCB on employee health and explores its other potential negative results, into which future studies can carry out in-depth research. Finally, this study reveals how HPL can lessen the negative impact of OCB on employee health, but there is still some room for improvement. Specifically, future research is advised to delve into the measures that can enable employees to engage in OCB with its adverse impact on them minimized; it can explore how to effectively reduce the impact of OCB from the perspectives of employees, such as their micro-breaks [64], and external environments, such as social support, or with the multiple factors taken into consideration simultaneously, such as organizations, superiors, and subordinates. Finally, how to promote OCB is another interesting research direction for future research. Previous research focus on the role of leadership behavior in promoting OCB, while overlooking leader’s cognitive underpinnings such as implicit followership theories. Future research could explore whether, why, and when leaders’ positive implicit followership theories promote followers’ actual OCB.

## 6. Conclusions

OCB has a potential detrimental effect on actors’ health. This is because OCB is positively related to citizenship fatigue, which in turn causes health complaint. Health-promoting leadership weakens the positive relationship between citizenship fatigue and health complaints, thus negatively moderating the indirect relationship between OCB and health complaints via citizenship fatigue.

## Figures and Tables

**Table 1:** Results of confirmatory factor analyses.

	χ^2^	df	χ^2^/df	RMSEA	SRMR	TLI	CFI
Hypothetical model (OCB, CF, HC, HPL)	128.36	48	2.67	0.08	0.05	0.92	0.94
Three-factor model (HC + HPL, OCB, CF)	541.64	51	10.62	0.22	0.18	0.52	0.63
Two-factor model (OCB + HC + HPL, CF)	504.71	53	9.52	0.20	0.19	0.58	0.66
Single-factor model (OCB + CF + HC + HPL)	751.99	54	13.93	0.25	0.17	0.36	0.47

Note: OCB stands for organizational citizenship behavior, CF stands for citizenship fatigue, HPL stands for health-promoting leadership, and HC stands for health complaints.

**Table 2:** Descriptive statistics, alpha coefficients, and correlations (*n* = 207).

	1	2	3	4	5	6	7	8	9
1. Gender	1								
2. Age	−0.10	1							
3. Dyadic tenure	0.02	0.37 ***	1						
4. Work hours per week	−0.23 **	0.08	0.12	1					
5. Negative emotions	0.04	−0.25 ***	−0.08	0.03	(0.85)				
6. OCB	−0.12	−0.14	−0.15 *	0.09	0.03	(0.96)			
7. Citizenship fatigue	−0.05	−0.07	0.10	−0.04	0.06	0.30 ***	(0.78)		
8. Health complaints	−0.05	−0.36 ***	−0.19 **	0.07	0.31 ***	0.39 ***	0.32 ***	(0.93)	
9. HPL	0.06	0.06	0.03	−0.07	0.35 **	−0.10	−0.04	−0.34 ***	(0.92)
Means	1.47	2.77	3.53	44.11	2.18	3.54	2.97	2.76	2.55
Standard deviations	0.50	0.96	3.99	9.05	0.94	0.65	0.63	0.92	1.00

Note: *n* = 207, *** *p* < 0.001, ** *p* < 0.01, * *p* < 0.05.

**Table 3:** Mediating role of citizenship fatigue and moderating effect of HPL.

Variable		Citizenship Fatigue	Health Complaints			
		Model 1	Model 2	Model 3	Model 4	Model 5
Control variable	Gender	−0.02	−0.06	−0.08	−0.06	−0.05
	Age	0.03	−0.17	−0.25	−0.17	−0.11
	Dyadic tenure	−0.11	0.25	−0.03	0.28	0.01
	Working hours per week	−0.008	−0.02	0.01	−0.02	−0.01
	Negative emotions	0.03	0.23 ***	0.22 ***	0.22 **	0.36 ***
Independent variable	OCB	0.29 ***	0.43 ***		0.34 ***	0.27 ***
Mediator	Citizenship fatigue			0.44 ***	0.30 ***	0.22 **
Moderator	HPL					−0.36 ***
Interaction term	Citizenship fatigue * HPL					−0.26 **
	R^2^	0.10 ***	0.32 ***	0.29 ***	0.36 ***	0.54 ***
	F	3.59	15.43	13.44	16.01	27.23

Note: *n* = 207, *** *p* < 0.001, ** *p* < 0.01, * *p* < 0.05.

**Table 4:** Examination of the moderated mediating effect (*n* = 207).

	Indirect Effect	Standard Error	95% Confidence Interval
Low HPL (−SD)	0.14	0.04	[0.07, 0.23]
High HPL (+SD)	−0.01	0.04	[−0.09, 0.06]
Difference	−0.15	0.06	[−0.29, −0.05]

Note: *n* = 207, low HPL has a mean minus 1 standard deviation while high HPL has a mean plus 1 standard deviation.

## Data Availability

Data are available from the authors upon reasonable request.
